# Impact of the COVID-19 pandemic lockdowns on manifest refraction in a young adult population: A large cohort study

**DOI:** 10.1007/s00417-025-06862-1

**Published:** 2025-06-04

**Authors:** Assaf Hilely, Dana Barequet, Aya Ekshtein, Liora Levian, Asaf Achiron, Yuval Kozlov, Oded Ben-Ari

**Affiliations:** 1 Division of Ophthalmology, Faculty of Medical and Health Sciences, Tel-Aviv Sourasky Medical Center, Tel Aviv University, 6 Weizmann Street, Tel Aviv, 64239 Israel; 2 Community Medical Services Division, Clalit Health Services, Tel Aviv, Israel; 3 The Israeli Air Force Aeromedical Center, Ramat-Gan, Israel; 4 Israel Defense Forces Medical Corps, Ramat-Gan, Israel; 5 Department of Military Medicine, Faculty of Medicine, The Hebrew University of Jerusalem, Jerusalem, Israel; 6 The Adelson School of Medicine, Ariel University, Ariel, Israel

**Keywords:** Refraction, Covid-19, Myopia, Lockdown, Spherical equivalent

## Abstract

**Purpose:**

To evaluate the impact of lockdowns during COVID-19 pandemic on manifest refraction in a young adult population.

**Methods:**

A retrospective, observational study of flight academy candidates evaluated between 2019 and 2023. Data collected included demographics, best corrected visual acuity, and manifest refraction. The cohort was divided into three groups with respect to the COVID-19 lockdown periods.

**Results:**

The study included 7,491 individuals with a mean age of 17.36 years (range 16–18), of whom 81% were male. Comparing refractive errors between the three groups, the highest rate of emmetropia, hyperopia and myopia were observed in the post-lockdown, during-lockdown group and pre-lockdown groups, respectively. Spherical equivalent (SE) significantly increased (a positive increase, indicating less myopia or more hyperopia) when comparing pre-lockdowns and during-lockdowns (mean difference = 0.100, *p* = 0.002), and between pre-lockdowns and post-lockdowns (mean difference = 0.119, *p* < 0.001). Multivariate regression analysis showed that both during-lockdowns and post-lockdowns periods were significantly associated with a positive shift in SE compared to the pre-lockdowns period (*p* = 0.005 and *p* = 0.001, respectively). Male gender was also a significant predictor for this hyperopic shift (*p* < 0.001).

**Conclusion:**

No increase in myopia was observed in healthy young adults, despite long periods of lockdowns and homeschooling during the Covid-19 pandemic.

## Abstract (highlights)

**Key messages:**

***What is known*:**

- Studies have shown myopic progression during the COVID-19 pandemic lockdowns in school-aged population.

***What is new*:**

- Our study showed no increase in myopia in a young and healthy adult population following the COVID-19 pandemic, despite extended periods of lockdowns and homeschooling.
- Combining four epithelial variables (Minimum, Max-Min, Midperipheral IT, and Midperipheral I) performed well in distinguishing topography normal eyes (AUC=0.896, sensitivity 76.3%, specificity 89.5%).
- Females exhibited a higher myopic tendency than males.

## Introduction

Myopia is a major health issue, as it is the most common cause of visual impairment worldwide [1]. Myopia is associated with ocular complications that may impact quality of life and place financial burden [2–4]. Duration and intensity of near-work activity and screen time, along with insufficient time for outdoor activity, are recognized as major risk factors for myopia development and progression [1, 5–7]. Other risk factors include age, gender, history of myopia in parents, and psycho-social stress.

Following the outbreak of the coronavirus disease (COVID-19) global pandemic, most nations imposed strict containment measures aimed to prevent further spread of the virus. These quarantines included school closure and limited outdoor activity, which led to an increase in the use of home digital screen devices. Many previous studies have shown that this increase in online schooling and use of electronic devices, along with decreased sunlight exposure, aggravated myopia progression, particularly in younger school-aged children [8–16]. Younger children’s refractive status may be more sensitive to environmental changes compared with that of older children, given that younger individuals are in a crucial period for the development of myopia. Nevertheless, studies from pre-COVID era [17, 18] and during the COVID pandemic [9, 12, 13, 19] have suggested that myopia may also continue progressing after puberty. As the world has entered the post-COVID-19 era, long-term data are available for assessing the actual effect of the pandemic period on myopia development. The purpose of our study was to evaluate the impact of the COVID-19 pandemic on manifest refraction in a young adult population.

## Methods

### Study design and participants

This was a retrospective analysis of electronic medical records of candidates who applied to the Israeli Air Force’s (IAF) aviator training program between January 2019 to April 2023.

Prior to enlistment at the age of 18 years, all candidates for the mandatory military service in Israel undergo a standard series of cognitive, psychological, and medical evaluations to assess their fitness for military service. These initial medical screenings are typically carried out by military physicians during the high school years, at least one year before graduation and enlistment at the age of 18 years. The standard evaluation process includes a detailed medical history review, urinalysis, vision tests, and a general physical examination. Further evaluations may be conducted for those with existing health issues. To qualify for the flight academy, candidates undergo additional, rigorous medical evaluations at the IAF Aeromedical Center (AMC). These assessments include a single comprehensive visual performance evaluation conducted by a trained optometrist. The tests performed encompass non-cycloplegic refraction, assessments of both uncorrected and best-corrected visual acuity for near and far vision, stereo acuity tests using the Titmus test, color vision assessments via the Ishihara test, and measurements of heterophoria for near and far. Data from these evaluations are collected and stored electronically.

### Refractive error and definitions

Noncycloplegic autorefraction was performed using the same autorefractometer for all participants in all study time periods (Tomey RT-7000, Nagoya, Japan, 2017). Spherical equivalent (SE) was calculated for each subject by combining the spherical power and one-half cylindrical diopters [D] obtained from autorefraction. Mild myopia was defined as a SE equal or more negative than − 0.5D but not more negative than − 3D. Moderate myopia was defined as a SE that is equal or more negative than − 3D but not more negative than − 6D. Severe myopia was defined as a SE that is −6D or more negative. Emmetropia was defined as SE greater than − 0.50D and less than + 0.5D, while hyperopia was defined as SE of + 0.5D or greater [4, 20].

### Study time periods

To thoroughly evaluate the impact of the pandemic on participants’ refractive outcomes, the sample was categorized based on the timing of their clinic visits in relation to Israel’s lockdown policy during the pandemic. The cut-off points were set at the start of the first lockdown (March 2020) and the end of the final lockdown in Israel (February 2021) [21].

Consequently, subjects were sorted into three subgroups based on the timing of their evaluations at the IAF AMC: those assessed before the first lockdown (January 1, 2019, to February 28, 2020), those assessed during the lockdowns (March 1 st, 2020, to February 28, 2021), and those assessed after the last lockdown period (March 1, 2021 to April 30, 2023).

To explore the influence of screen time exposure during the lockdowns, we employed a dose-response assessment approach. Each participant in the during-lockdowns group was assigned a numerical score reflecting time elapsed from the initiation of the first lockdown until their assessment date. Participants assessed at the end of the last lockdown received the highest scores, which indicated a longer duration of screen time exposure.

### Data analysis

Statistical analyses were conducted using R (version 4.2.2) and RStudio (version 2023.06.0 Build 421). The SE of the right and left eyes in this sample showed a strong correlation (Pearson’s correlation coefficient = 0.880, *p* < 0.0001). Therefore, subsequent analyses included only the right eye of the subjects. Also, p-value < 0.05 was considered statistically significant for all tests.

Descriptive statistics, such as means and standard deviations (SDs), were calculated for continuous variables, and frequencies were calculated for categorical variables. ANOVA tests were performed to analyze the differences in continuous variables to independent variables with three or more categories. Tukey post-hoc tests were performed for each significant ANOVA test. Pearson’s correlation coefficients were utilized to determine the relationship between continuous variables.

Pearson’s chi-square analysis was conducted to evaluate the relationship between categorical variables. For comparisons of categories with more than two levels, post hoc pairwise chi-square tests were performed with a Bonferroni adjustment for multiple comparisons.

Lastly, a multivariate linear regression model was implemented to determine the impact of the independent variables on the change in SE.

### Ethical considerations

This study was approved by the Israeli Defense Force (IDF) Institutional Review Board (IRB approval number 2275 − 2022).

## Results

### Participants demographics and distribution of refractive errors

A total of 7,491 individuals were included in this analysis, with a mean age of 17.36 ± 0.48 years (range: 16–18 years) at the time of evaluation. The majority of participants in this study were males (81.04%).

**Fig. 1:**
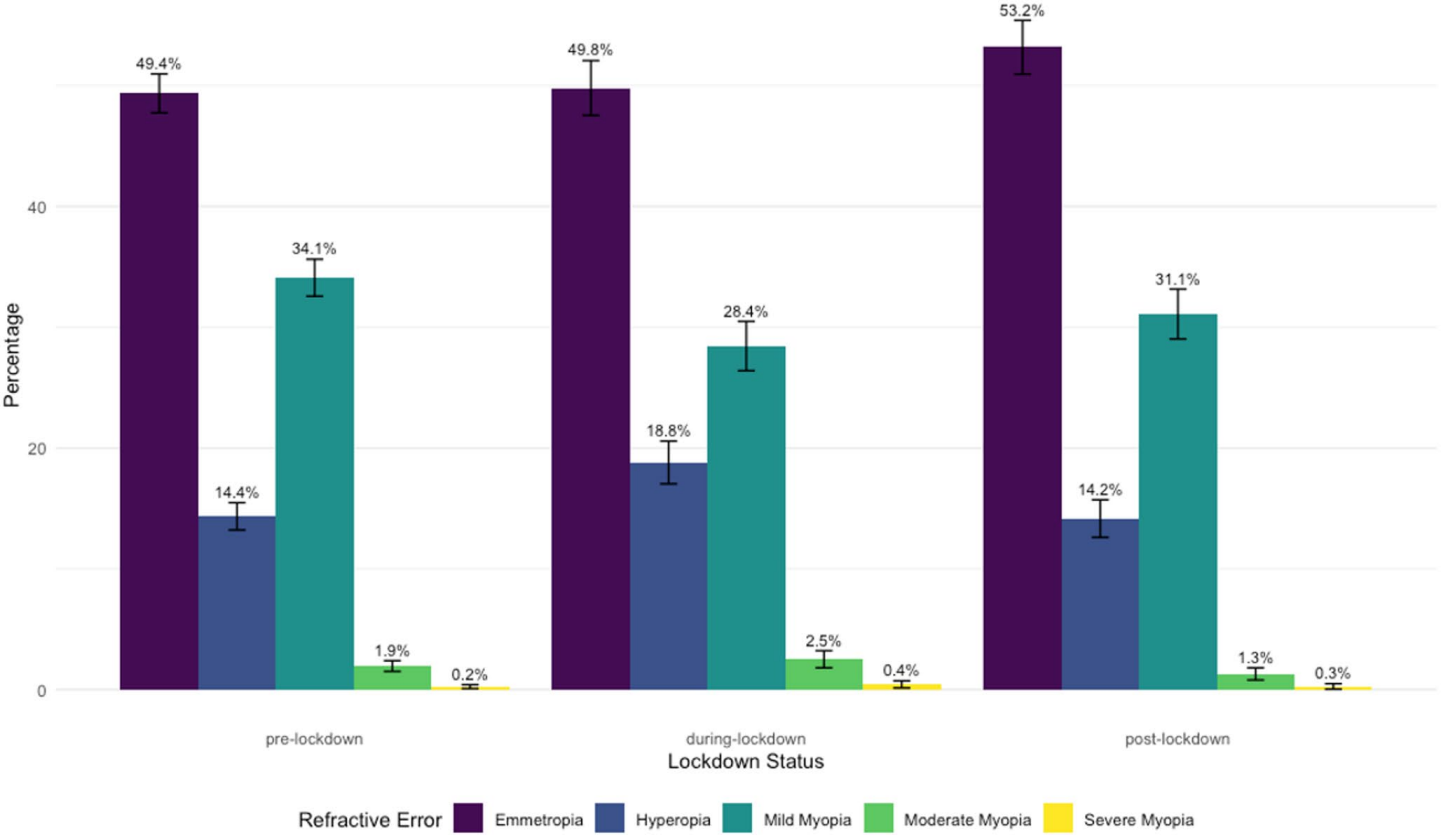
Prevalence of refractive error groups (Hyperopia, Emmetropia, and Myopia) across the study time periods

We further categorized the participants into three groups based on the three study time periods: pre-lockdowns, during-lockdowns, and post-lockdowns. Table 1 exhibits baseline characteristics and SE of each subgroup. Both age and gender distributions demonstrated significant differences across these subgroups (*p* < 0.0001). The prevalence of refractive error groups (hyperopia, emmetropia, and myopia) across the study time periods are demonstrated in Fig. 1.

**Table 1:** Baseline characteristics and spherical equivalent metrics across the study time periods

Period	Age [years] (Mean ± SD, Range)	Sex [%] (M: F, %)	Refractive Group (*N*, % of Total)	SE [Diopter] (Mean ± SD, Range)
Pre-lockdowns (*N* = 3,693)	17.3 ± 0.46 (16–18)	79.30:20.7	**Emmetropia** *N* = 1,823, 49.36%	0.00 ± 0.23 (−0.38, 0.48)
			**Hyperopia** *N* = 530, 14.35%	0.84 ± 0.61 (0.50, 4.88)
			**Mild Myopia** *N* = 1259, 34.09%	−1.30 ± 0.65 (−2.88, −0.50)
			**Moderate Myopia** *N* = 72, 1.95%	−3.72 ± 0.77 (−5.75, −3.00)
			**Severe Myopia** *N* = 9, 0.24%	−6.90 ± 0.54 (−7.50, −6.13)
During-lockdowns (*N* = 1,871)	17.4 ± 0.50 (16–18)	82.00:18.00	**Emmetropia** *N* = 932, 49.81%	0.01 ± 0.23 (−0.38, 0.39)
			**Hyperopia** *N* = 352, 18.81%	0.87 ± 0.60 (0.50, 4.50)
			**Mild Myopia** *N* = 532, 28.43%	−1.18 ± 0.61 (−2.88, −0.50)
			**Moderate Myopia** *N* = 47, 2.51%	−4.01 ± 0.86 (−5.88, −3.00)
			**Severe Myopia** *N* = 8, 0.43%	−8.84 ± 3.68 (−16.75, −6.00)
Post-lockdowns (*N* = 1,927)	17.5 ± 0.50 (16–18)	83.30:16.7	**Emmetropia** *N* = 1025, 53.19%	0.00 ± 0.23 (−0.38, 0.38)
			**Hyperopia** *N* = 273, 14.17%	0.86 ± 0.53 (0.50, 5.25)
			**Mild Myopia** *N* = 599, 31.08%	−1.08 ± 0.58 (−2.88, −0.50)
			**Moderate Myopia** *N* = 25, 1.30%	−3.90 ± 0.84 (−5.50, −3.00)
			**Severe Myopia** *N* = 5, 0.26%	−8.30 ± 1.68 (−11.25, −7.25)

### Influence of lockdown time periods on refractive error prevalence

Our analysis revealed a significant impact of the lockdown period on the prevalence of different refractive errors (*p* < 0.0001). Post hoc analyses were conducted to assess the effect on each refractive category across the periods.

Significant differences were observed in the Emmetropia group when comparing pre-lockdowns to both during-lockdowns and post-lockdowns periods (*p* < 0.0001), with the highest rate of emmetropia in the post-lockdowns period (53.19%). For the Hyperopia group, a significant increase in prevalence was observed during the lockdown period (18.81%) compared to the pre- and post-lockdowns periods (*p* < 0.0001 and *p* < 0.0016, respectively). Additionally, both Mild and Moderate Myopia categories showed statistically significant differences across the periods (*p* < 0.0001), with the highest rate of mild myopia in the pre lockdowns period (34.09%).

### Univariate analysis of SE changes

Our univariate analysis aimed to directly investigate the influence of lockdowns on SE, without categorizing into refractive error groups. We found that SE significantly differed between pre-lockdowns and during-lockdowns (mean difference = 0.100, *p* = 0.002), and between pre- and post-lockdowns (mean difference = − 0.119, *p* < 0.001). In both cases, the SE during and after the lockdowns was higher, indicating that subjects assessed in these periods had less myopic tendency on average in relation to the pre-lockdowns period.

We also examined the effect of screen time exposure during the lockdowns in a dose-response measure. Our findings suggest a weak but significant positive correlation between the exposure score and SE values (*r* = 0.09, *p* < 0.0001), indicating that subjects assessed later in the lockdowns period had less myopic results.

### Multivariate regression analysis of SE changes

Finally, a multivariate linear regression analysis was conducted to account for potential confounding variables such as age and gender on the SE results in relation to the assessment onset. A summary of the two models is presented in Table 2. The first model focused on the influence of the three study time periods while the second model focused on time exposure during the lockdowns.

**Table 2:** Multivariate linear regression models for spherical equivalent

Variables	Estimate	Std.Coef	*p*-value	95% CI
Model 1 - Influence of the study time periods on SE change				
During-Lockdowns Period	0.085	0.034	0.005	[0.025, 0.146]
Post-Lockdowns Period	0.099	0.040	0.001	[0.038, 0.159]
Age (years)	0.026	0.0121	0.310	[−0.024, 0.078]
Sex (male)	0.349	0.125	< 0.001	[0.284, 0.413]
Model 2 - Influence of exposure duration (during lockdown only)				
Exposure Time (days)	0.0009	0.072	0.001	[0.0003, 0.001]
Age (years)	−0.049	−0.0203	0.395	[−0.163, 0.064]
Sex (male)	0.404	0.126	< 0.001	[0.253, 0.554]

In the first model, both during-lockdowns and post-lockdowns periods were significantly associated with an increase in SE compared to the pre-lockdowns period (*p* = 0.005 and *p* = 0.001, respectively), when adjusted for age and gender. Age was not a significant predictor, but being male was associated with a significant increase in SE (*p* < 0.001).

In the second model, which only included the subjects during lockdowns, higher exposure was positively associated with increased SE, with an estimated increase of 0.0009 units for each additional unit of exposure time (*p* = 0.001). Age did not significantly impact SE, but being male was strongly associated with higher SE values (*p* < 0.0001).

## Discussion

This large-based population study investigated the refractive changes prior to, during and following the COVID-19 pandemic with respect to the lockdown periods in a young and healthy adult population. Overall, our analysis showed an increase in SE during and post-lockdown periods, signifying less myopic tendency.

Several studies have shown myopic progression during the COVID-19 pandemic lockdowns. However, it should be mentioned that this tendency was shown only with respect to younger children and was explained by the extended near-work periods and restricted outdoor activity [8–16]. Teenagers and young adults were found to have less myopia, as was shown in this study. Yang et al. [14], for example, revealed in a meta-analysis a significant myopic shift during the COVID-19 era among children between 5 and 11 years. However, this significant change was not observedamong theolder school aged children between ages of 11 and 18 years. Another study by Chang et al. [11] also found that younger participants are more susceptible to myopic progression during lockdown compared to teenagers. Zhou et al. [15] showed that while mean SE declined in grades 3–5 and grades 7–8 during and following the COVID era, in higher grades, the change was not significant and even drifted toward hyperopia in some cases.

There are several explanations for this discrepancy. First, the refractive mechanism of young children is thought to be more susceptible to lifestyle changes brought about by the pandemic. Also, lifestyle changes induced by the pandemic lockdowns were not as obvious in teenagers as they were in young children, for older children had already been exposed to digital devices for a long time before COVID-19. Another possibility which may behypothesized in respect to our cohort, is that although the quarantines aimed to stop the spread of the virus and confined most people to their homes, there were actually no restrictions on individual outdoor sport activities including walking and running, outdoor gym facilities and hiking. As our studyconsisted of young, healthy, athletic, and motivated young adults, it may very well be that the lack of face-to-face education left this population with extra time for athletic workout, resulting in a positive refractive result.

A few studies were conducted in the adult population and included groups of university students [17, 18], laboratory employees [22], and higher-school graders [10, 12]. These studies showed, indeed, a significant myopic progression during the study period, however, it was attributed to the fact that all participants were committed to near work and therefore at risk of increasing myopia.

Our study also showed a significant difference in refractive errors between males and females (*p* < 0.001), with females exhibiting lower SE than males, indicating a greater myopic tendency in this group. The multivariate analysis revealed gender to be the only significant predictor of increased SE. Studies addressing gender disparity in myopia progression during the COVID-19 outbreak are rare, yet some have shown that females had higher rates and earlier development of myopia than males [9, 11, 15]. The reason is not fully understood, however, it has been postulated that earlier hormonal changes affecting the ocular structure in females may play a role in this difference.

The limitations of our study include its retrospective nature and its relatively homogenous population comprised of young, healthy air force academy candidates. Despite the cohort being large, it may not accurately represent the age-matched population. In addition, we did not collect information regarding other risk factors for myopia development such as screen or outdoor times and parental myopia. It should also be noted that manifest refraction was used for defining myopia. Although this parameter is useful and effective for screening and monitoring myopia in a large-scale population, it is less accurate than cycloplegic refraction [20]. Finally, although the calculated effect of exposure time on SE change is small in magnitude, it is important to consider its’ cumulative effect; there may be a considerably greater impact relevant to the long duration of lockdowns in the study.

## Conclusion

In this study, no increase in myopia was observed in a young and healthy adult population following the COVID-19 pandemic, despite extended periods of lockdowns and homeschooling.

## Data Availability

The datasets used and/or analyzed during the current study are available from the corresponding author on reasonable request.
